# 6-Chloro-*N*-methyl-5-nitro-*N*-phenyl­pyrimidin-4-amine

**DOI:** 10.1107/S1600536811037664

**Published:** 2011-09-20

**Authors:** Fuqiang Shi, Li-Hong Zhu, Li Mu, Long Zhang, Ya-Feng Li

**Affiliations:** a School of Chemical Engineering, Changchun University of Technology, Changchun 130012, People’s Republic of China; b School of Bioscience and Technology, Changchun University, Changchun 130022, People’s Republic of China

## Abstract

In the title compound, C_11_H_9_ClN_4_O_2_, the dihedral angle between the aromatic rings is 79.67 (8)°. π–π stacking between centrosymmetrically related pairs of pyrimidine rings occurs along [100] [centroid–centroid separations = 3.4572 (8) and 3.5433 (7) Å].

## Related literature

For a related structure, see: Shi *et al.* (2011 ▶).
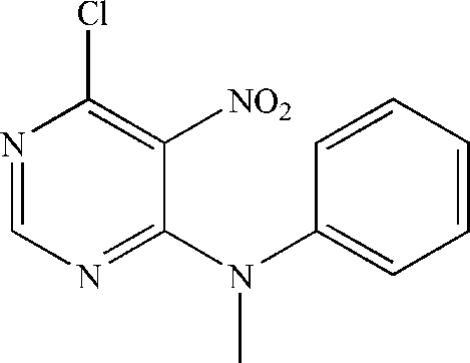


## Experimental

### 

#### Crystal data

C_11_H_9_ClN_4_O_2_
*M* *_r_* = 264.67
Triclinic, 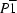

*a* = 6.8980 (14) Å
*b* = 8.9282 (18) Å
*c* = 11.427 (2) Å
α = 73.76 (3)°
β = 86.80 (3)°
γ = 84.21 (3)°
*V* = 672.0 (2) Å^3^
*Z* = 2
Mo *K*α radiation
μ = 0.28 mm^−1^
*T* = 293 K
0.44 × 0.38 × 0.13 mm

#### Data collection

Rigaku R-AXIS RAPID diffractometer
Absorption correction: multi-scan (*ABSCOR*; Higashi, 1995 ▶) *T*_min_ = 0.885, *T*_max_ = 0.964
5925 measured reflections
2730 independent reflections
1742 reflections with *I* > 2σ(*I*)
*R*_int_ = 0.030

#### Refinement

*R*[*F* ^2^ > 2σ(*F* ^2^)] = 0.049
*wR*(*F* ^2^) = 0.154
*S* = 1.07
2730 reflections
164 parameters
H-atom parameters constrained
Δρ_max_ = 0.25 e Å^−3^
Δρ_min_ = −0.19 e Å^−3^

### 

Data collection: *PROCESS-AUTO* (Rigaku, 1998 ▶); cell refinement: *PROCESS-AUTO*; data reduction: *CrystalStructure* (Rigaku/MSC, 2002 ▶); program(s) used to solve structure: *SHELXS97* (Sheldrick, 2008 ▶); program(s) used to refine structure: *SHELXL97* (Sheldrick, 2008 ▶); molecular graphics: *DIAMOND* (Brandenburg, 2000 ▶); software used to prepare material for publication: *SHELXL97*.

## Supplementary Material

Crystal structure: contains datablock(s) I, global. DOI: 10.1107/S1600536811037664/ng5229sup1.cif

Structure factors: contains datablock(s) I. DOI: 10.1107/S1600536811037664/ng5229Isup2.hkl

Supplementary material file. DOI: 10.1107/S1600536811037664/ng5229Isup3.cml

Additional supplementary materials:  crystallographic information; 3D view; checkCIF report

## Figures and Tables

**Fig. 1.**
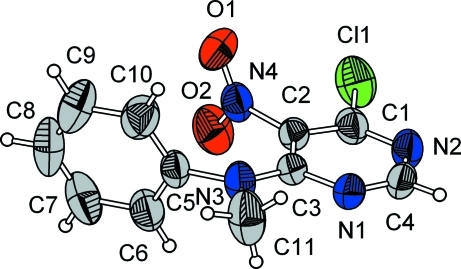
The title compound, C11H9ClN4O2, with the atom-labelling scheme. Displacement ellipsoid are shown at the 50% probability level.

**Fig. 2.**
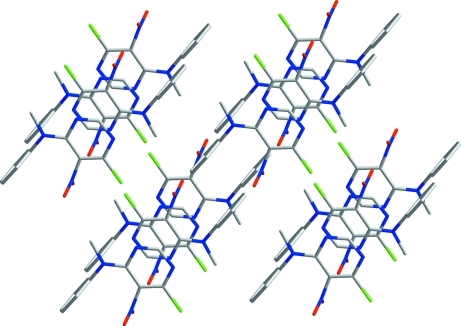
Aromatic π-π stacking between centrosymmetrically related pairs of pyrimidine rings along 100].

## supplementary crystallographic information

### Comment

Here, the crystal structure of 6-chloro-*N*-methyl-5-nitro-*N*-phenylpyrimidin-4-amine, the precursor of 6-chloro-*N*-methyl-*N*-phenylpyrimidine-4,5-diamine (Shi *et al.*, 2011) is determined by X-ray single crystal diffraction.

In the structure of (I) (Fig. 1), the dihedral angle between the aromatic rings is 79.667 (81)°. Uninterrupted aromatic π-π stacking between centrosymmetrically related pairs of pyrimidine rings occurs along with [100] direction [centroid – centroid separation = 3.4572 (8)Å or 3.5433 (7)Å].

### Experimental

To a solution of 4,6-dichloro-5-nitro-pyrimidine (2.08 g, 10.8 mmol), and triethylamine (13.0 mL, 0.55 mmol) in anhydrous THF (25 mL) was added a solution of *N*-methylbenzylamine (0.85 mL, 10.8 mmol) in anhydrous THF (15 mL) slowly. The reaction mixture was stirred at room temperature overnight. The reaction mixture was concentrated in vacuo, diluted with water, and extracted with EtOAc. The organic phase was washed with 1N HCl, brine, dried over anhydrous MgSO_4_, and concentrated in vacuo to yield the crude product as a solid. Purification by recrystallization from methanol provided the desired pure product, 6-chloro-*N*-methyl-5-nitro-*N*-phenylpyrimidin-4-amine (yellow solid, 1.85g, 64.7%, 130.3-131.4 °C). ^1^H NMR (CDCl_3_, 400 Hz), δ: 8.51 (s, 1H), 7.393-7.37(m, 3H), 7.17-7.15(m, 2H), 3.57 (s, 3H); ^13^C NMR (CDCl_3_, 100 Hz), δ: 156.6, 153.9, 152.4, 142.2, 129.8, 128.6, 126.3, 41.7. ES-MS: 265.0 [(M + H^+^)].

### Refinement

All H atoms were located from difference Fourier maps. H atoms attached to C atoms were treated as riding [C—H = 0.93–0.96 Å, *U*_iso_(H) = 1.2U_eq_(aromatic carbon) and *U*_iso_(H) = 1.5U_eq_(methyl carbon)].

### Crystal data

**Table:**

C_11_H_9_ClN_4_O_2_	*Z* = 2
*M**_r_* = 264.67	*F*(000) = 272
Triclinic, *P*1	*D*_x_ = 1.308 Mg m^−^^3^
Hall symbol: -P 1	Mo *K*α radiation, λ = 0.71073 Å
*a* = 6.8980 (14) Å	Cell parameters from 500 reflections
*b* = 8.9282 (18) Å	θ = 3.4–27.5°
*c* = 11.427 (2) Å	µ = 0.28 mm^−^^1^
α = 73.76 (3)°	*T* = 293 K
β = 86.80 (3)°	Block, colorless
γ = 84.21 (3)°	0.44 × 0.38 × 0.13 mm
*V* = 672.0 (2) Å^3^	

### Data collection

**Table:**

Rigaku R-AXIS RAPID diffractometer	2730 independent reflections
Radiation source: fine-focus sealed tube	1742 reflections with *I* > 2σ(*I*)
graphite	*R*_int_ = 0.030
Detector resolution: 10.00 pixels mm^-1^	θ_max_ = 27.5°, θ_min_ = 3.4°
ω scans	*h* = −8→7
Absorption correction: multi-scan (*ABSCOR*; Higashi, 1995)	*k* = −10→10
*T*_min_ = 0.885, *T*_max_ = 0.964	*l* = −14→14
5925 measured reflections	

### Refinement

**Table:**

Refinement on *F*^2^	Primary atom site location: structure-invariant direct methods
Least-squares matrix: full	Secondary atom site location: difference Fourier map
*R*[*F*^2^ > 2σ(*F*^2^)] = 0.049	Hydrogen site location: inferred from neighbouring sites
*wR*(*F*^2^) = 0.154	H-atom parameters constrained
*S* = 1.07	*w* = 1/[σ^2^(*F*_o_^2^) + (0.0827*P*)^2^ + 0.0097*P*] where *P* = (*F*_o_^2^ + 2*F*_c_^2^)/3
2730 reflections	(Δ/σ)_max_ < 0.001
164 parameters	Δρ_max_ = 0.25 e Å^−^^3^
0 restraints	Δρ_min_ = −0.19 e Å^−^^3^

### Special details

**Table:**

Geometry. All e.s.d.'s (except the e.s.d. in the dihedral angle between two l.s. planes) are estimated using the full covariance matrix. The cell e.s.d.'s are taken into account individually in the estimation of e.s.d.'s in distances, angles and torsion angles; correlations between e.s.d.'s in cell parameters are only used when they are defined by crystal symmetry. An approximate (isotropic) treatment of cell e.s.d.'s is used for estimating e.s.d.'s involving l.s. planes.
Refinement. Refinement of *F*^2^ against ALL reflections. The weighted *R*-factor *wR* and goodness of fit *S* are based on *F*^2^, conventional *R*-factors *R* are based on *F*, with *F* set to zero for negative *F*^2^. The threshold expression of *F*^2^ > σ(*F*^2^) is used only for calculating *R*-factors(gt) *etc*. and is not relevant to the choice of reflections for refinement. *R*-factors based on *F*^2^ are statistically about twice as large as those based on *F*, and *R*- factors based on ALL data will be even larger.

### Fractional atomic coordinates and isotropic or equivalent isotropic displacement parameters (Å^2^)

**Table:**

	*x*	*y*	*z*	*U*_iso_*/*U*_eq_	
Cl1	0.27001 (12)	0.38397 (9)	0.55435 (6)	0.0897 (3)	
C1	0.2612 (3)	0.1851 (3)	0.52481 (19)	0.0585 (5)	
C2	0.2496 (3)	0.1772 (2)	0.40780 (17)	0.0483 (5)	
C3	0.2386 (3)	0.0133 (2)	0.38625 (17)	0.0485 (5)	
C4	0.2470 (3)	−0.1014 (3)	0.59315 (19)	0.0655 (6)	
H4	0.2468	−0.1895	0.6599	0.079*	
N1	0.2346 (3)	−0.1270 (2)	0.48527 (16)	0.0600 (5)	
N2	0.2610 (3)	0.0465 (3)	0.62119 (16)	0.0694 (6)	
C5	0.2510 (3)	0.1072 (2)	0.16239 (18)	0.0544 (5)	
C6	0.4329 (4)	0.1522 (3)	0.1186 (2)	0.0716 (7)	
H6	0.5428	0.1063	0.1631	0.086*	
C7	0.4528 (5)	0.2692 (4)	0.0051 (3)	0.0954 (9)	
H7	0.5759	0.3000	−0.0227	0.114*	
C8	0.2935 (6)	0.3371 (4)	−0.0638 (2)	0.1037 (11)	
H8	0.3077	0.4122	−0.1381	0.124*	
C9	0.1135 (6)	0.2917 (4)	−0.0205 (3)	0.1034 (11)	
H9	0.0043	0.3376	−0.0656	0.124*	
C10	0.0897 (4)	0.1749 (3)	0.0930 (2)	0.0817 (8)	
H10	−0.0337	0.1445	0.1202	0.098*	
C11	0.2102 (5)	−0.1969 (3)	0.2719 (3)	0.0965 (10)	
H11A	0.3338	−0.2570	0.2895	0.145*	
H11B	0.1728	−0.1948	0.1917	0.145*	
H11C	0.1136	−0.2440	0.3307	0.145*	
N4	0.2395 (3)	0.3422 (2)	0.31108 (16)	0.0611 (5)	
N3	0.2279 (3)	−0.0184 (2)	0.27815 (15)	0.0610 (5)	
O1	0.0799 (3)	0.4051 (2)	0.27365 (17)	0.0885 (6)	
O2	0.3903 (3)	0.4091 (2)	0.27659 (17)	0.0889 (6)	

### Atomic displacement parameters (Å^2^)

**Table:**

	*U*^11^	*U*^22^	*U*^33^	*U*^12^	*U*^13^	*U*^23^
Cl1	0.1161 (6)	0.1002 (6)	0.0686 (4)	−0.0261 (4)	0.0009 (4)	−0.0440 (4)
C1	0.0513 (12)	0.0778 (15)	0.0475 (11)	−0.0073 (9)	−0.0013 (9)	−0.0187 (10)
C2	0.0444 (10)	0.0555 (12)	0.0418 (10)	−0.0054 (8)	0.0014 (8)	−0.0081 (9)
C3	0.0462 (11)	0.0536 (12)	0.0419 (10)	−0.0013 (8)	0.0020 (8)	−0.0082 (9)
C4	0.0575 (13)	0.0777 (16)	0.0451 (12)	0.0008 (10)	−0.0005 (9)	0.0073 (11)
N1	0.0608 (11)	0.0606 (11)	0.0494 (10)	−0.0012 (8)	0.0025 (8)	−0.0022 (8)
N2	0.0645 (12)	0.0960 (15)	0.0422 (10)	−0.0040 (10)	−0.0054 (8)	−0.0101 (10)
C5	0.0695 (14)	0.0575 (12)	0.0366 (10)	−0.0065 (9)	0.0008 (9)	−0.0137 (9)
C6	0.0695 (16)	0.0932 (18)	0.0524 (13)	−0.0116 (12)	0.0033 (11)	−0.0199 (12)
C7	0.103 (2)	0.123 (2)	0.0616 (16)	−0.0383 (18)	0.0260 (16)	−0.0237 (16)
C8	0.158 (3)	0.105 (2)	0.0433 (14)	−0.033 (2)	0.0002 (18)	−0.0056 (14)
C9	0.129 (3)	0.107 (2)	0.0651 (17)	−0.0076 (19)	−0.0391 (19)	−0.0022 (16)
C10	0.0740 (17)	0.102 (2)	0.0656 (15)	−0.0096 (13)	−0.0142 (13)	−0.0141 (14)
C11	0.170 (3)	0.0617 (16)	0.0635 (16)	−0.0234 (16)	0.0113 (17)	−0.0244 (12)
N4	0.0828 (14)	0.0553 (11)	0.0452 (10)	−0.0049 (9)	0.0051 (9)	−0.0154 (8)
N3	0.0842 (13)	0.0542 (11)	0.0436 (9)	−0.0103 (8)	0.0035 (8)	−0.0115 (8)
O1	0.0992 (14)	0.0837 (13)	0.0688 (11)	0.0162 (10)	−0.0197 (10)	−0.0035 (9)
O2	0.1063 (15)	0.0742 (12)	0.0829 (13)	−0.0353 (10)	0.0259 (11)	−0.0125 (9)

### Geometric parameters (Å, °)

**Table:**

Cl1—C1	1.905 (2)	C6—H6	0.9300
C1—C2	1.366 (3)	C7—C8	1.375 (5)
C1—N2	1.408 (3)	C7—H7	0.9300
C2—C3	1.560 (3)	C8—C9	1.368 (4)
C2—N4	1.573 (3)	C8—H8	0.9300
C3—N3	1.349 (3)	C9—C10	1.431 (4)
C3—N1	1.436 (2)	C9—H9	0.9300
C4—N1	1.324 (3)	C10—H10	0.9300
C4—N2	1.456 (3)	C11—N3	1.633 (3)
C4—H4	0.9300	C11—H11A	0.9600
C5—C6	1.380 (3)	C11—H11B	0.9600
C5—C10	1.388 (3)	C11—H11C	0.9600
C5—N3	1.488 (3)	N4—O1	1.226 (2)
C6—C7	1.430 (4)	N4—O2	1.242 (3)
			
C2—C1—N2	119.2 (2)	C6—C7—H7	119.4
C2—C1—Cl1	119.29 (17)	C9—C8—C7	118.4 (2)
N2—C1—Cl1	121.46 (16)	C9—C8—H8	120.8
C1—C2—C3	118.29 (17)	C7—C8—H8	120.8
C1—C2—N4	113.29 (18)	C8—C9—C10	121.4 (3)
C3—C2—N4	128.35 (16)	C8—C9—H9	119.3
N3—C3—N1	110.90 (18)	C10—C9—H9	119.3
N3—C3—C2	127.02 (16)	C5—C10—C9	120.0 (3)
N1—C3—C2	122.06 (17)	C5—C10—H10	120.0
N1—C4—N2	128.60 (19)	C9—C10—H10	120.0
N1—C4—H4	115.7	N3—C11—H11A	109.5
N2—C4—H4	115.7	N3—C11—H11B	109.5
C4—N1—C3	112.86 (19)	H11A—C11—H11B	109.5
C1—N2—C4	118.93 (18)	N3—C11—H11C	109.5
C6—C5—C10	118.8 (2)	H11A—C11—H11C	109.5
C6—C5—N3	121.0 (2)	H11B—C11—H11C	109.5
C10—C5—N3	120.1 (2)	O1—N4—O2	120.9 (2)
C5—C6—C7	120.2 (2)	O1—N4—C2	118.81 (18)
C5—C6—H6	119.9	O2—N4—C2	120.25 (19)
C7—C6—H6	119.9	C3—N3—C5	120.02 (17)
C8—C7—C6	121.2 (3)	C3—N3—C11	120.80 (17)
C8—C7—H7	119.4	C5—N3—C11	118.93 (17)
